# Repeated intravenous thrombolysis in early recurrent ischemic stroke: a case report of an elderly low weight female

**DOI:** 10.1007/s10072-023-07060-9

**Published:** 2023-09-19

**Authors:** Feifei Chen, Xiangting Chen, Qian Zhang, Siyuan Wen, Changqing Zhou

**Affiliations:** Department of Neurology, Bishan Hospital of Chongqing Medical University, No. 9 Shuangxing Avenue, Bishan District, Chongqing, 402760 China

## Introduction

Guidelines in many countries advise against intravenous thrombolysis (IVT) for the patient with a history of stroke within 3 months for the presumed higher risk of intracranial hemorrhage. However, some observational studies indicated that IVT may be effective and safe in some patients. This article reports a successful case of repeated IVT with low-dose alteplase in an 80-year-old low weight female patient with recurrent ischemic stroke within 9 days after the index stroke.

## Case report

An 80-year-old female, whose BMI was 22.5 kg/m^2^ (height: 149 cm, weight: 50 kg), was admitted to hospital for emotional anxiety and general malaise for 4 years and aggravating for 1 month. She was diagnosed with “coronary heart disease” by coronary angiography 6 years ago, but she did not take antiplatelet drugs regularly. After admission, cardiology consultation recommended anticoagulation treatment for atrial fibrillation, but the patient refused anticoagulation treatment for the risk of hemorrhage.

**Fig. 1:**
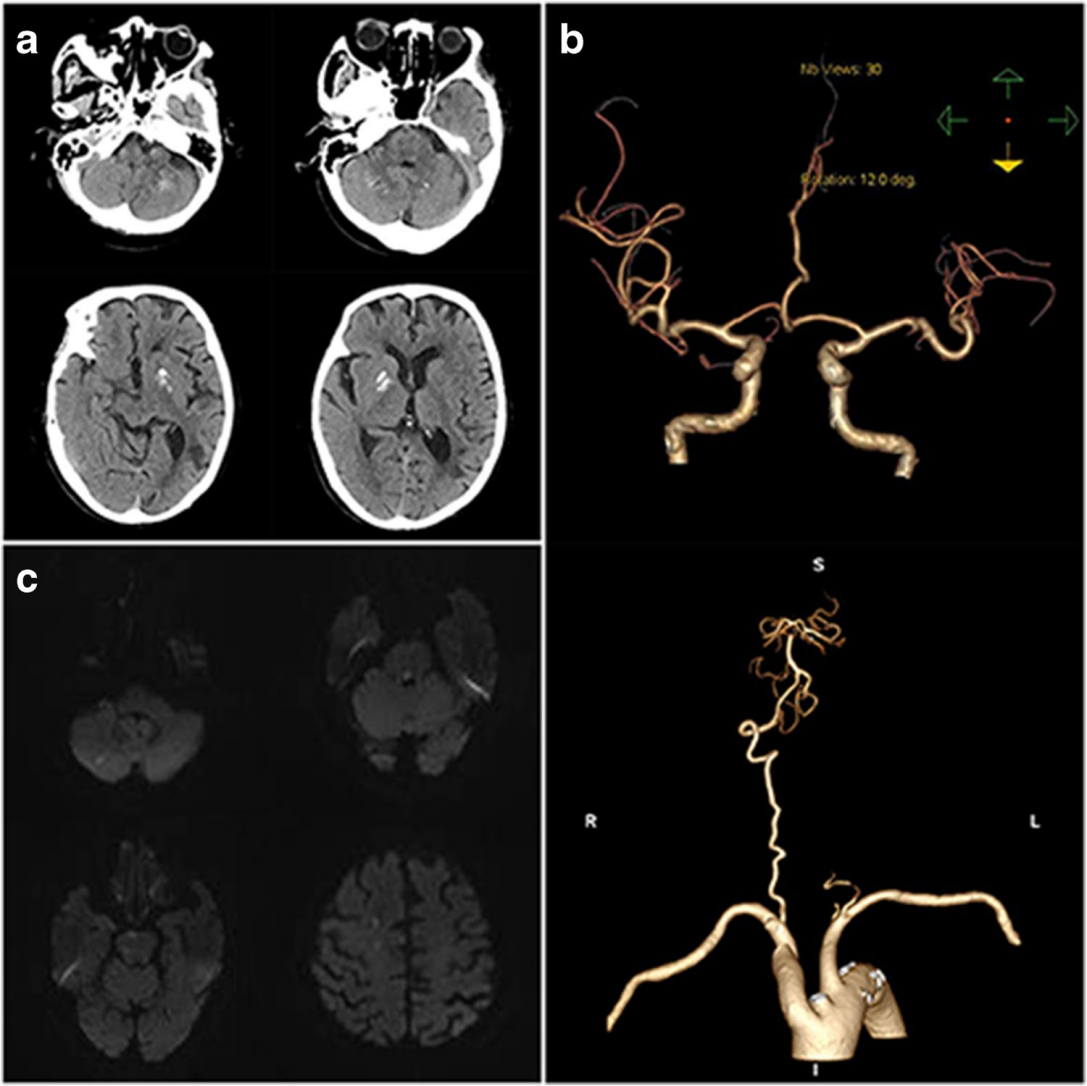
Imaging of patient with the first stroke. **a** Head CT on 3rd day after admission (before thrombolysis): no cerebral hemorrhage. **b** Head and neck CTA on 3rd day after admission: the left vertebral artery and the terminal of the basilar artery were not visualized. **c** Brain MRI on day 3rd day after admission: acute cerebral infarction scattered in the right frontal lobe, pons, and right cerebellar hemisphere

On the 3rd day after admission, the patient suddenly suffered from aphasia and left-sided hemiplegia. The expanded National Institutes of Health Stroke Scale (e-NIHSS) score was 10, and modified Rankin Scale (mRS) score was 5. The patient underwent emergency head computed tomography (CT) examination, which excluded cerebral hemorrhage (Fig. 1a). The emergency head and neck CT angiography (CTA) showed that the left vertebral artery and the terminal of the basilar artery were not visualized (Fig. 1b). A diagnosis of acute cerebral embolism was made and IVT with alteplase (0.9 mg/kg, 45 mg) was performed. The brain magnetic resonance imaging (MRI) (after thrombolysis) confirmed scattered acute cerebral embolisms in the right frontal lobe, pons, and right cerebellar hemisphere (Fig. 1c). On the 4th day after admission, the aphasia was improved, the deviated mouth was recovered, and the muscle strength of the left limb was graded 4 + . The e-NIHSS score was only 1, and mRS score was 1. There were many dermal ecchymoses on both thighs, and indobufen 0.1 g bid was given for anti-platelet treatment. The follow-up head CT did not show any cerebral hemorrhage.

**Fig. 2:**
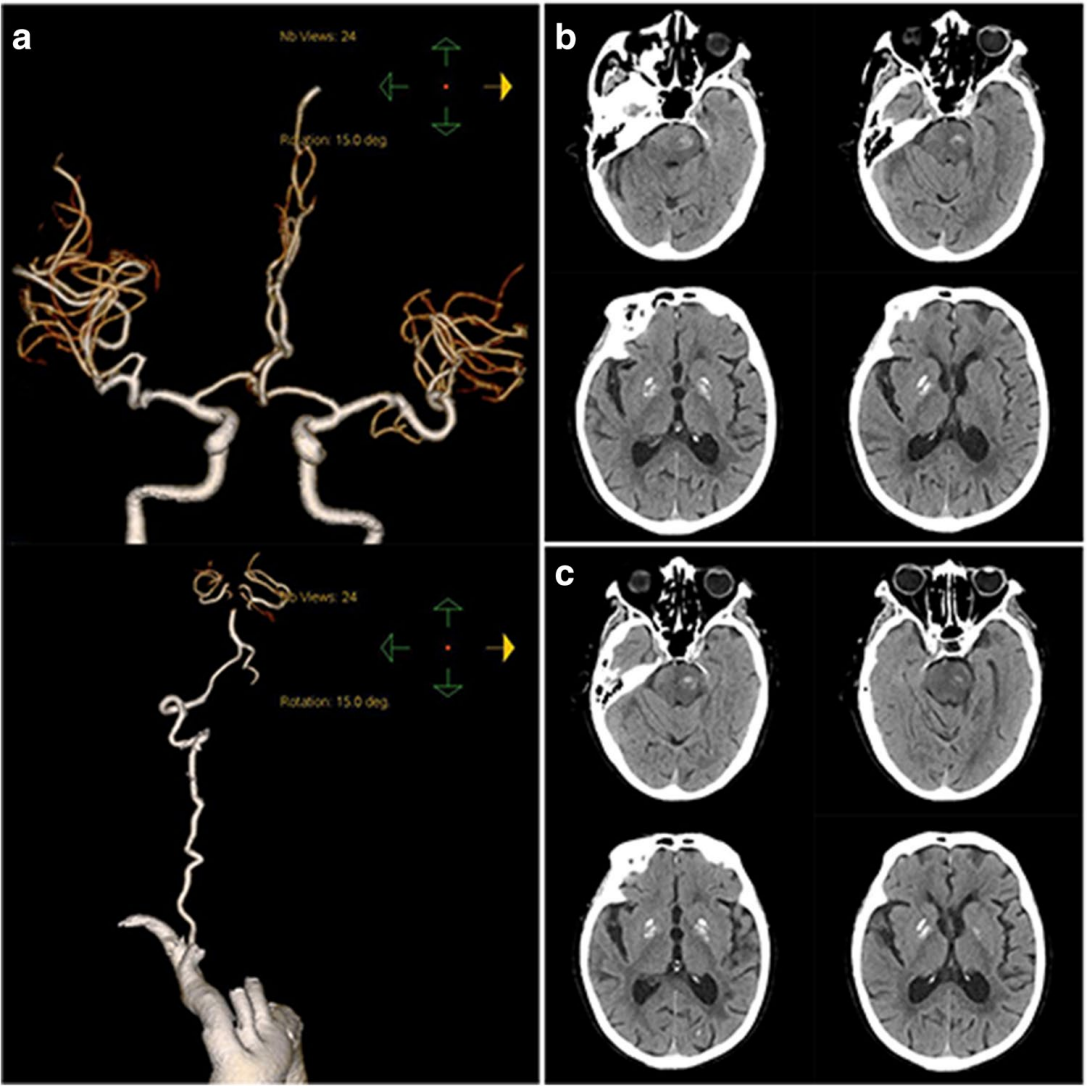
Imaging of patient with the second stroke. **a** Head and neck CTA on the 12th day after admission: the left vertebral artery and the terminal of the basilar artery were not visualized as before; the basilar artery and the origin of bilateral posterior cerebral arteries were not visualized locally. **b** Head CT on the 13th day after admission: small patchy hemorrhages in the left occipital lobe and pons. **c** Head CT on the 15th day after admission: compared with the CT on the 13th day after admission, the hematoma did not change significantly

On the 12th day after admission, the patient suddenly developed slurred speech, skewed mouth angle, and left-sided hemiplegia again. The diagnosis of recurrent acute cerebral embolism in the vertebrobasilar territory was made. The e-NIHSS score was 14, and mRS score was 5. The emergency head and neck CTA showed left vertebral artery and the terminal of the basilar artery were not visualized as before. The basilar artery and the origin of bilateral posterior cerebral arteries were not visualized locally (Fig. 2a). After communication with the family, a second IVT was performed with low-dose alteplase (0.6 mg/kg, 30 mg). On the 13th day after admission, the symptoms were significant improved and the e-NIHSS score was 2 and mRS score was 1. Follow-up head CT showed a small patchy hemorrhage in the left occipital lobe and pons (Fig. 2b). On the 15th day after admission, most symptoms were disappeared and the e-NIHSS score was 2, and mRS score was 1. The head CT showed that the cerebral hemorrhage had not enlarged (Fig. 2c).

At the time of discharge 3 weeks after admission, the patient was slightly slurred, the skewed corners of the mouth improved, the muscle strength of the left limb was 4 + grade, and the NIHSS score was 2, and mRS score was 1.

## Discussion

There are many stroke patients who recur within 3 months. And there are still some patients who do not have these high-risk factors of symptomatic intracranial hemorrhage (sICH) except recurring stroke within 3 months. If thrombolysis is not carried out in patients who recur stroke within 3 months, some patients will lose valuable treatment opportunities. There was no evidence that alteplase was less effective in patients with high predictive risk of sICH than in those with lower risk. Repeated IVT may be safe and effective in patients with small infarct volumes and robust clinical improvement, when recurrent stroke occurs within 3 months. Therefore, the history of stroke within 3 months is not an absolute contraindication to IVT.

IVT was limited to treating acute ischemic stroke patients under 80 years old. However, there is no report about whether age is an independent risk factor for sICH in secondary thrombolysis within 3 months. A review [1] showed that the important risk factors for sICH after IVT are history of diabetes, higher systolic blood pressure, male sex, race (Asian), etc. Therefore, if the elderly patients (> 80 years old) combine with other risk factors mentioned above, the risk of sICH may increase.

Low body weight increases the risk of ischemic stroke and cerebral hemorrhage [2]. The risk of hemorrhage increased when weight was less than 60 kg and significantly increased when weight was less than 50 kg [2]. Similarly, patients with a BMI of more than 25 kg/m^2^ had half the odds of a poor prognosis compared with those with a BMI of less than 25 kg/m^2^. Thus, the dose of alteplase should be carefully chosen when administering IVT to patients with low body weight.

The higher incidence of sICH in patients with atrial fibrillation (AF) after IVT treatment is an important reason for the poor prognosis. A study [3] showed that acute ischemic stroke patients with atrial fibrillation (AF) more frequently had poor outcome after IVT therapy compared with those without AF. The reason for the higher risk of bleeding in stroke patients with AF may be that patients appear to be more likely to have large or old thrombi, which are resistant to IVT [3]. As for the etiology of the recurrence to our patient, we were unable to specify the stroke etiology classification [4] for the refusal of the patient and her family to undergo a digital subtraction angiography (DSA) and the multiple causes of atrial fibrillation, coronary heart disease, advanced age, etc. Therefore, we cannot rule out that the patient was a cardiogenic cerebral embolism, nor can we determine whether the patient was a large atherosclerotic cerebral embolism. In other words, the stroke etiology classification of the patient was unclear.

There are many factors associated with sICH after IVT. We chose the iScore [5], one of the alteplase related sICH risk scoring systems which indicate that age, NIHSS score, and atrial fibrillation were associated with sICH after IVT, to grade our patient. For this scoring system of sICH, iScore ≤ 139 means low risk, iScore 140–179 means medium risk, and iScore ≥ 180 means high risk. And our patient with a score of 200 (age 80, sex 0, stroke severity 65, stroke subtype 30, risk factor 10, comorbid condition 0, preadmission disability 15, glucose on admission(6.1 mmol/L) 0) had a high risk of sICH.

A systematic review [6] showed that the rates of sICH just ranged from 0 to 16.6% in patients treated with low dose alteplase in all studies included, and 48 to 76.92% of them also achieved good functional outcomes, which indicated that use of low-dose alteplase in ischemic stroke patients resulted in significantly lower incidence of SICH.

In accordance with the guideline [7], for endovascular treatment of acute ischemic stroke, endovascular treatment is effective in patients with recurrent stroke. At the same time, endovascular treatment may be more important in patients at high risk of recurrence [8–10]. In addition, mechanical thrombectomy is an effective treatment option, especially for eligible patients with recurrent event [11, 12]. Moreover, multiple studies have shown good clinical outcomes with repeat endovascular therapy in patients with recurrent stroke [13]. However, for our patient, the reasons why we did not perform endovascular treatment for large vessel occlusion (LVO) can be listed as follows. Firstly, the endovascular approach is very important for the patient. At the time of the first stroke, we suggested endovascular treatment to the patient and his family, but the patient declined for the related risks. Secondly, the patient’s brain magnetic resonance imaging (MRI) examination showed scattered acute cerebral infarction in the right frontal lobe, brainstem, and right cerebellar hemisphere, with small and scattered infarct foci, but no obvious acute infarct foci were seen in the territory of the tip of basilar artery. Thirdly, at the time of recurrent stroke, according to the patient’s head and neck CTA, most of the left vertebral artery was not visualized and the basilar artery and the beginning of the posterior cerebral arteries bilaterally were not visualized, which are consistent with the CTA findings at the first stroke. This confirms the fact that the unrevealed vertebral and basilar arteries may be old lesions and not responsible for the acute cerebral infarction, and therefore, the patient was not considered for mechanical thrombectomy for a further acute cerebral infarction.

Therefore, we performed an secondary thrombolytic therapy in a patient aged over 80 low body weight with recurrence of ischemic stroke within 9 days, and the neurological function was significantly improved, with NIHSS score from 14 to 2. Given the high risk of sICH, we administered a low dose of alteplase (0.6 mg/kg) for IVT during the second thrombolysis to avoid severe sICH in our patient. Although the patient still had a small amount of hemorrhage, she achieved a good functional outcome. This verified we chose an appropriate treatment strategy.

In conclusion, for elderly and low weight patients with small infarct volume and good clinical outcome in the index stroke, low-dose alteplase (0.6 mg/kg) thrombolysis can be considered for recurrent acute ischemic stroke within 3 months. More studies are required to define the appropriate dosage of repeated intravenous thrombolysis, especially in elderly and low weight patients.

## Data Availability

The datasets used and analysed during the current study available from the corresponding author on reasonable request.
